# Confinement and scarcity: maternal rearing conditions and offspring performance in a predatory mite of biological control relevance

**DOI:** 10.1093/cz/zoag058

**Published:** 2026-08-25

**Authors:** Lenin Dzibakwe Chari

**Affiliations:** Department of Biological, Environmental and Earth Sciences, University of South Carolina, 471 Iniversity Pkwy, Aiken, SC 29801, USA

Predatory mites in the family Phytoseiidae are among the most widely deployed biological control agents for the control of small arthropod pests such as spider mites, thrips, and whiteflies. To be effective agents, they are commercially mass-produced before they can be released. However, rearing conditions used to mass-produce these predators are not well researched, but to optimize rearing, it would be beneficial to know optimal conditions for aspects such as cage size, prey density, and impacts of rearing generation after generation in enclosed arenas. Gong et al. (2026) ask a simple but consequential question: does it matter how cramped or how well-fed a mother mite is, not just for her, but for her daughters?

The authors experimented on *Amblyseius herbicolus*, a mite with great biocontrol potential that has females that reproduce without mating (thelytokous; Fig. 1). Using a 2 × 2 design, the authors varied cage/cell size (large vs. small) and food supply (30 vs. 60 prey eggs per day), then tracked how mothers and their offspring performed under each combination. The result is a rare demonstration of how spatial and nutritional stress in captivity ripple forward through maternal effects.

**Figure 1:**
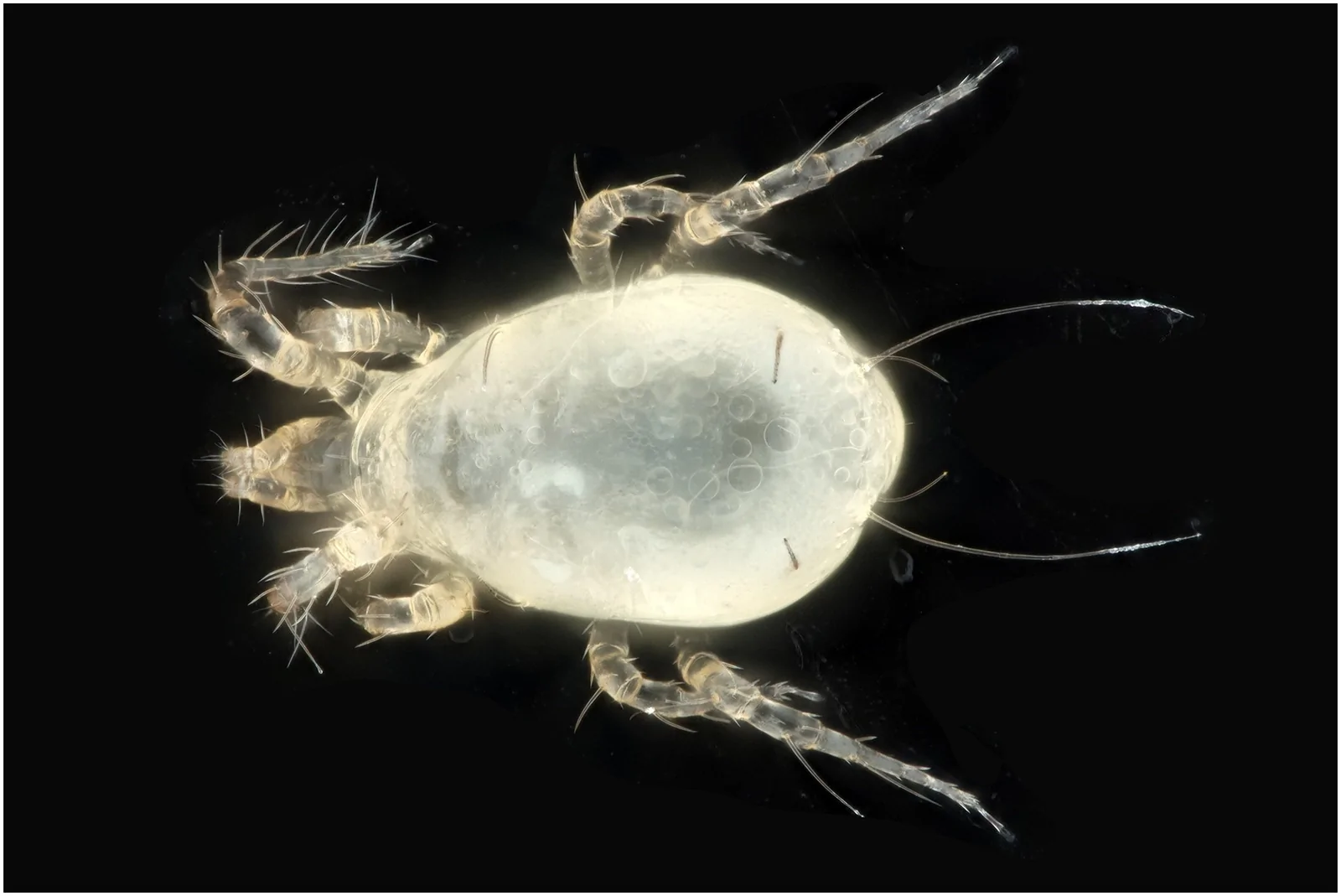
Dorsal view of adult female predatory mite (*Amblyseius herbicolus*) that has potential for being a biological control agent of pests such as broad mites, spider mites, and thrips. Image courtesy of Gong et al. (2026).

Within the maternal generation, the costs of confinement were clear. Mothers reared in small cells ate substantially more than those in large cell, yet that extra intake did not improve survival, development time, or adult size. The authors interpret this as an indication that energy was being expended on stress rather than growth. Prey scarcity compounded the problem as survival dropped sharply in small cells when food was also limited, though abundant prey buffered much of that cost. Reproductive investment shifted too, with high prey availability paradoxically producing smaller eggs from mothers in large cells, hinting at condition-dependent adjustments in per-egg allocation.

The results in the offspring generation were very different. Daughters of mothers reared under the toughest conditions, with small cells and scarce prey, did not inherit their mothers’ disadvantage. Instead, they showed higher survival and larger adult size than daughters of mothers reared with abundant food. This is a compensatory rather than a simply transmitted maternal effect as adversity in one generation appears to prime the next for resilience. As a possible explanation for this behavior, the authors suggest a principle called hormesis (Calabrese and Mattson 2017), where a mild dose of stress can sometimes trigger a beneficial response rather than simply causing harm. The authors suggest something similar may be happening across generations here as a mother's mild confinement stress may prime her offspring to perform better, rather than just passing along harm.

For a field devoted to optimizing biocontrol production, the implications are practical as well as conceptual. Mass-rearing protocols are typically tuned to maximize the performance of the generation currently in the tray, but this study suggests that the history of the rearing line, for example, how much space and food previous generations experienced, can shape the quality of the mites eventually released into a crop. Although the paper does not go as far as making this assertion, it is worth asking whether a colony reared too comfortably for too long risks producing individuals less primed for the unpredictability of a real field environment.

Beyond its applied value, this paper is a useful reminder that “control” and “constant” are not the same thing in laboratory ecology. Rearing conditions are experimental variables in their own right, and their legacy does not stop at the individual. This study shows how it can be carried into the next generation through mechanisms we are only beginning to understand mechanistically. The manuscript's careful factorial design and 2-generation tracking make it a template other groups working with phytoseiids, and invertebrate rearing systems more broadly, could readily adopt.

It is a pleasure to see this work recognized as a cover article, and I am glad to introduce it to readers here.
